# Enhanced Labeling to Promote Consumption of Nutrient Dense Foods and Healthier Diets

**DOI:** 10.3390/foods13213377

**Published:** 2024-10-24

**Authors:** Charles Benbrook, Robin Mesnage

**Affiliations:** 1 Benbrook Consulting Services, Port Orchard, Washington, DC 98367, USA; 2 Buchinger Wilhelmi Development & Holding GmbH, 88662 Überlingen, Germany; robin.mesnage@buchinger-wilhelmi.com; 3 Department of Nutritional Sciences, School of Life Course Sciences, Faculty of Life Sciences and Medicine, King’s College London, London SE1 9NH, UK

**Keywords:** nutrients, food labeling, nutrient density, health claims, dietary choice

## Abstract

**Background/Objectives:** Efforts are underway worldwide to design and deploy food labeling systems that provide consumers with the information needed to shift dietary patterns toward nutrient dense, healthier foods. Despite a compelling need for progress, worrisome public health trends persist that are rooted in the popularity of unhealthy, heavily processed foods. **Methods:** The nutrition and health-related content on the packaging of nine common foods sold in the US and Europe is analyzed and compared. The current scope of nutrient-specific messaging is characterized, including messages highlighting health-related benefits stemming from the mix and levels of mostly macronutrients in food products. **Results:** An average of 6.9 unique nutrition-related messages appear on the packaging of nine US food products, while EU food products contain an average of 5.0. Messaging around the ingredients in food products accounts for the largest share, e.g., “100% whole grain”, “Vegan”, and “No artificial preservatives”. The macronutrients of fat, fiber, cholesterol, salt, sugar, and protein are the focus of most messaging around health benefits. The degree of food processing and essential vitamin, mineral, and phytochemical micronutrients receive little or no attention, despite their importance in positive health outcomes. **Conclusions:** Current nutrition-related labeling fails to inform consumers of the enormous differences in the contribution of food products in meeting nutritional needs. Existing metrics and rating systems do not effectively account for the critical relationship between nutrient density and caloric content. Existing metrics and systems do not reflect the impacts of processing on food nutritional quality in ways that provide consumers meaningful information. New concepts, metrics, and label elements are described that could promote healthier dietary patterns. Clear and mandatory nutrition labeling could begin shifting market share toward healthier options, and this could trigger and guide changes in manufactured food recipes that make brand-name products healthier, benefiting all consumers.

## 1. Introduction

Opportunities abound to enhance public health through changes in food quality and dietary choices. Nearly one-half of all Americans have one or more chronic health conditions rooted in food and beverages, including 40% of school-age children [1,2]. Only about 25% of individuals aged 17–24 are regarded as prepared for military basic training [3]. There has been an “alarming decline” in food nutritional quality [4,5,6]. Some 90% of the estimated USD 4.3 trillion in annual health care costs in the US is triggered or made worse by poor food quality and diet-related disease [1,2,3,4,5,6]. Serious problems have surfaced with the conceptual foundation and scientific integrity of the US Dietary Guidelines, with a focus on their failure to adequately account for associations between food nutritional quality and chronic disease [7,8,9]. Healthier food and diets will benefit individuals, families, and communities, yet unwelcome trends persist in most countries.

The gap between current and optimal food quality and dietary-intake patterns is enormous, especially in the United States (US) [10,11], including for children [12]. As a result, the US Food and Drug Administration (FDA) is working on a new front-of-pack labeling scheme with the goal of providing shoppers clearer guidance toward healthier choices [13]. The FDA’s “Notice” seeking public comments on the definition of healthy food and the design of new front-of-package nutrition labeling appeared in the Federal Register on 26 January 2023 [14]. It states that: “FDA seeks to improve dietary patterns in the United States to help reduce the burden of nutrition-related chronic diseases and advance health equity as nutrition-related diseases are experienced disproportionally by certain racial and ethnic minority groups and those with lower socioeconomic status”.

The FDA’s effort to enhance nutrition labeling could help set the stage for “food is medicine”, a key action item emerging from a 2022 White House Conference on Hunger, Nutrition, and Health [15]. The US government is also promoting the notion of food is or as medicine via a number of initiatives [16,17].

Existing food labeling systems in the US and the European Union (EU) focus predominantly on a food product’s macronutrients: calories, fat, cholesterol, carbohydrates, sugar, fiber, protein, and salt (see Section 3 “Results” and Supplementary Tables S1–S18). In the US, a standardized Nutrition Facts panel appears on the back of food packaging (and sometimes a side panel; see examples in Supplemental Tables S1–S18: Front-of-Pack and Back-of-Pack Nutrition and Health-Related Information, Claims, and Messaging on Nine Food Products in the US and the EU). These panels report mostly macronutrient content per serving of food and convey information on serving size and the number of servings in a package. The amount in grams and percent of the Daily Value (DV) is also reported for selected vitamins, calcium, iron, potassium, and some other micronutrients. Similar macronutrient content information appears on product labels in the EU but is typically expressed per 100 g or 100 mL of beverages, and sometimes also per serving. Regulations have been adopted in the EU governing acceptable ways to convey nutrition information to consumers [18] and an effort is underway to improve and harmonize food labeling within Europe [19]. 

Food packaging contains other food ingredient, nutrient, and nutrition-related information in both the US and EU that fall into three general categories: (1) information about what ingredients are, or are not in a food product (e.g., whole grain, vegan, no bioengineered content), (2) potential or implied health benefit(s) (e.g., promotes heart health, low cholesterol, reduced sodium, healthy bones), and (3) established linkages between the nutrient content in a product and some specific health outcomes (e.g., qualified health claims in the US [20]). Most nutrient- and nutrition-related information on food labels in the US and EU focuses on macronutrient content and impacts on health outcomes.

Raw food ingredients, additives, and other processing agents are included in manufactured-food recipes and must be included in a product’s ingredient list in both the US and EU. Given the current focus in biomedical journals and lay media on the impacts of food processing on metabolic syndrome and other adverse health outcomes, food companies and consumers are increasingly focused on a product’s degree of processing [21,22,23]. The NOVA food processing system is named after the word “novel” in Portuguese, and first appeared in the original NOVA paper by Monteiro et al. [24] describing a new system to place foods into one of four categories:

1. Unprocessed or minimally processed foods;
2. Processed culinary ingredients;
3. Processed foods;
4. Ultra-processed foods.

The NOVA system is now used widely to characterize the degree of processing in food labeling and marketing applications, as well as in epidemiological research exploring associations between the degree of food processing and diverse health outcomes [25]. In Europe, Nutri-Score values are now encouraged in several countries and appear on many food products [26,27]. Like NOVA in the US, Nutri-Score values are also beginning to be used in epidemiological studies (e.g., [28,29]).

Questions persist, however, over whether existing systems provide a sufficiently accurate basis for delineating healthy from unhealthy food choices [30,31,32,33,34,35,36,37,38,39]. The EU’s food labeling regulations also do not quantify the degree of processing or provide clear guidance to consumers who seek to avoid ultra-processed food [18]. As part of the European Farm to Fork strategy, the European Commission was called upon and expected to propose a harmonized, mandatory front-of-pack nutrition labeling scheme by the end of 2023 but has yet to do so. Responsible authorities in the EU are struggling to work through the same technical, messaging, and political issues and undercurrents that are proving challenging for the US FDA [33,35,36,37,38].

Research led by Serge Hercberg from the University of Sorbonne and University Paris Cité developed and tested “Nutri-Score 2.0”, an enhanced nutrition label that includes a prominently displayed warning about ultra-processed foods [28]. The study involved over 21,000 participants who assessed products with the standard Nutri-Score, the modified version, or no label. Nutri-Score 2.0 improved consumers’ understanding of nutrient content and the degree of processing. Most participants found the Nutri-Score 2.0 label credible and helpful, with 88% supporting its use on packaging. 

A review by Devaux et al. [36] compares four front-of-pack labeling systems, including Nutri-Score, Keyole, Nutri-Repere, and Nutri-Couleurs. Compared to the three other systems, Nutri-Score was deemed superior in reducing caloric intake and improving public health. Nutri-Score led to an estimated 3% reduction in calories. While even such modest progress is welcomed, change sufficient to meaningfully alter disturbing food and diet-driven public health trajectories will almost certainly require more effective interventions.

A system designed to support nutrition-health labeling on food packages (front, back, top, bottom, and sides) that targets consumers is inevitably going to differ from a system designed to support epidemiological research exploring the health outcomes stemming from food nutritional quality, degree of processing, and food choices and dietary patterns. Ideally, the core concepts and metrics used to support nutrition labeling and conduct food-health research will share common roots, but operational details will invariably diverge.

To provide consumers with reliable guidance on how to shift dietary patterns in ways likely to enhance health outcomes, it is essential to quantify the nutritional quality of one category of food product compared to others (e.g., breakfast cereal to fresh fruit), as well as between one brand of a particular food compared to competing brands (Cheerios versus Cheerios with Honey or Raisin Bran).

Herein, we argue that such a system should quantify nutritional quality on the basis of a recommended serving of food, as opposed to on the basis of an equal number of grams or ounces of food, or a set number of calories (e.g., 100 calories). A focus on serving sizes will provide nutritional quality guidance to consumers in a way aligned with the information on Nutrition Facts panels that are also expressed per serving. Previously, Benbrook, Mesnage, and co-authors submitted comments to the FDA on its “Proposed rule” for new FOP labeling that identified the serious problems with the FDA’s proposed approach and recommended alternative metrics for incorporation in a hypothetical labeling system called NuCal [40].

**Figure 1:**
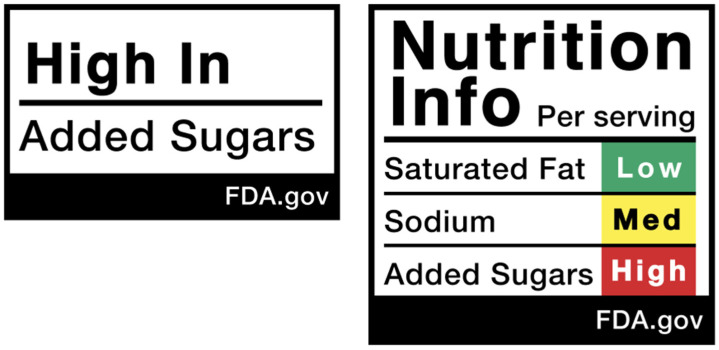
Graphics displaying macronutrient composition under consideration for front-of-pack nutrition labeling by the US FDA.

The FDA is developing front-of-pack nutrition labeling options that convey graphically the most important information on macronutrient content that is currently contained in Nutrition Fact panels. Current options under consideration by the FDA appear in Figure 1.

Among many challenges, both technical and political, the FDA must decide the macronutrient “take home” messages to feature in simple yet hopefully compelling front-of-pack graphics [39,41]. But to date, the FDA has not publicly discussed ways to provide consumers comparable information on many other nutrients essential in achieving positive public health outcomes, nor has the FDA addressed what to communicate, if anything, about the degree and impacts of food processing.

In this paper, we assess the nutrient- and nutrition-related information on the packaging of a representative sample of foods and brands for sale in the US and EU. We critique current nutrition-related messaging and identify other key nutritional quality attributes of food that are not addressed adequately, or at all. Suggestions are made to improve the content on food packaging based on the FDA’s stated purpose in its ongoing review of FOP options. The impacts over time of improved, clearer, and mandatory nutritional labeling on the recipes used by food manufacturers, and hence also on the nature and degree of processing, is emphasized as a secondary benefit of food labeling that shifts purchase decisions toward healthier products.

## 2. Materials and Methods

Nine common food products sold in the US and EU were selected for the assessment of their label content: Cheerios, oatmeal, Pringles, whole wheat bread, plant-based ground round (i.e., plant-based hamburger), whole milk, almond milk, Thousand Island salad dressing, and ketchup. All the products entail a degree of processing (milk is pasteurized and fortified) and several are or would likely be classified as ultra-processed under the NOVA system (e.g., Pringles, Thousand Island dressing).

We use the nutritional quality metric described herein to calculate values for a selection of foods ranging from heavily processed to whole, fresh food forms. Details of the nutrient profiling system used to generate these values are described below and in Supplemental File: Methodology and Data Sources in Calculating Nutritional Quality Values for Specific Foods.

### 2.1. Nutrient and Nutrition-Health Messaging on Labels

We captured all nutrient- and nutrition-health-related information on food package labeling, including information on the front-of-pack (FOP), back-of-pack (BOP), and if any, side and top-and-bottom panels. Quantitative indicators are reported of the overall content of nutrient-specific and nutrition health-related label messages appearing on product packaging.

On some food products, the same or similar nutrition health-related information appears more than once. The number of repeated messages on each product’s packaging is reported and taken into account when computing the number of unique nutrient content and nutrition health-related information items. Pictures of the nutrition-related content on food product packaging for each of the nine foods from both the US and EU markets appear in Supplemental File Tables S1–S18, along with the content and number of messages. There are two Supplemental Tables for each food. One compares the front-of-pack content of the food product sold in the US to the same food product sold in Europe. The second Supplemental Table for each food covers all nutrient- and nutrition-related label content on the back-of-pack, and side and/or bottom-top panels (if any).

Information items on food packaging that has relevance in communicating the nutrient content, nutritional quality, and healthfulness of a given food product are placed in one of three categories (see Table 1):

- Content Information;
- Implied Health Benefit, and;
- Qualified Health Claim.

“Content Information” refers to one or more of the ingredients in a food product. Such messaging links an ingredient in the food product to some presumptively positive attribute. For example, messaging related to gluten content, whether the food is vegan, or contains “whole” ingredients, fall in the “Content Information” category, whereas claims regarding recycling, impacts on rainforests, or fair trade and treatment of workers are excluded for lack of relevance to nutritional quality.

Two characteristics apply to messages in the “Implied Health Benefit” category. First, such messages refer to a relatively higher or lower level of one or more specific nutrients or ingredients in a food product, compared to competing brands and/or other foods. Second, the higher or lower level of certain ingredients and/or nutrients in the product is generally thought to promote a positive health outcome in light of government-set, recommended daily intakes. Nutri-Score [26,42] or NOVA [23,24] system ratings would fall in this category since the scores in both systems are based on various nutrient-related attributes or shortcomings in a food product that can impact health outcomes. Messages in this category do not communicate a generally accepted association between a health benefit and the contents of the food product.

The third category includes label information and messaging that communicates a specific linkage between an altered level of one or more nutrients in a food product and some specific and named health outcome (e.g., CVD, osteoporosis). In the US, the FDA has established a rigorous, data-rich process for approving or denying petitions that propose a new “Qualified Health Claim” on food packages, as well as in advertising and other channels of communication [20]. Petitions may be submitted to the FDA by food companies or other entities (e.g., a commodity group representing tomato growers, a research institute).

Such health claims are “qualified” by specifying the circumstances in which an association between some identified attribute in a food product (e.g., 30% more calcium) is expected to promote a positive health outcome (stronger bones) or lower the risk of a negative one (osteoporosis). Qualifying statements often include the circumstances in which the outcome is likely to occur (e.g., “in conjunction with a healthy lifestyle” or “when part of a healthy diet”).

For each of the nine food products, we counted the total number of nutrient and health-related messages on product packaging within each of the three categories in Table 1. The average number of messages by category is reported in Table 2 below.

### 2.2. Quantifying and Ranking the Nutritional Quality of Food Products

Our preferred framing of “nutritional quality” in the context of a food labeling system intended to improve public health outcomes is the degree to which a serving of food meets essential nutrient needs, while not taking up a disproportionate share of daily caloric space (e.g., 2000 calories per FDA guidance). Such a metric takes account of the portion of essential nutrients in a serving of food relative to applicable Recommended Daily Intakes for each nutrient, and the share of daily caloric intake fulfilled by that serving of food. Such a metric is part of a hypothetical food labeling system called NuCal that is described in the HHRA comments to the FDA [40]).

A nutrient profiling system is required to quantify the NuCal metric for a given serving of food [40,43]. There are 27 nutrients in the NuCal system: eleven vitamins, eight minerals, protein, fiber, antioxidant activity as measured by total ORAC, lutein + zeaxanthin, linoleic acid, linolenic acid, lycopene, and choline. RDA-equivalent daily intake levels regarded as necessary to avoid health problems and serving sizes are from US government documents, or in the absence of a government-set value, recommended intakes advanced by public or private sector authorities with recognized expertise in the nutritional sciences.

The basic, single-nutrient metric in one serving of food is a ratio: nutrient (mg)/RDA (mg) (or equivalent). This is expressed as a percentage.

The NuCal value for one serving of fresh orange is calculated as follows:

NuCal Value = NQI/% Caloric Space

where

NQI is the Nutritional Quality Index, i.e., the % of daily needs across 27 nutrients that is satisfied by the nutrients in one medium-sized orange (NQI = 7.54%);
% Caloric Space is the share of a 2000 calorie diet taken up by the calories in one medium-sized orange (62 calories; 62/2000, or 3.1%)

We have calculated the NuCal values for 196 common foods (see Supplemental File: NuCal and Nutritional Quality Index (NQI) Values for 196 Foods in Three Zones Along the Nutritional Quality Continuum). The foods include dozens of single-ingredient fresh and whole foods; cereals; grain-based products; meat, poultry, and fish; and several multi-ingredient foods (pepperoni pizza, a Big Mac and fries). Values for some foods are reported based on different food forms (fresh grapes versus raisins or grape juice) and methods of cooking (fresh, fried, boiled). For the 196 foods, NQI values are calculated per 100 g of food, per 100 calories, and per typical serving size. The later NQI value based on the grams or ounces in a serving of food is used in the NuCal system.

A medium orange delivers 7.5% of the total nutrients needed to sustain health based on RDAs or equivalent intake benchmarks, and the weights assigned to nutrients in the NQI (see details on weighting in Supplemental File: Methodology and Data Sources in Calculating Nutritional Quality Values for Specific Foods). The NuCal value for a medium orange pr serving is 2.4 (7.5%/3.1%). Accordingly, a medium-sized orange delivers 2.4 times more progress toward satisfying daily nutrient needs than the caloric space taken up by the calories in a medium-sized orange.

Several decisions and assumptions have to be made in order to calculate NuCal values for any given food. The way each of these issues is addressed in calculating NuCal values for specific foods assessed in this paper is noted in in each of the six items addressed below. Regardless of the metrics chosen as the basis of future nutritional quality labeling systems, a method to deal with the following six computational issues will need to be developed and vetted:

1. What essential nutrients should be included (e.g., the 27 in NuCal, or some other set).
2. How to set a widely applicable, recommended daily intake level for each nutrient deemed essential (e.g., in NuCal, RDAs, other benchmark intakes sufficient to sustain good health for adult women, or some other population cohort).
3. Weights assigned to individual nutrients in the calculation of overall nutritional quality; e.g., a higher weight on a nutrient that is routinely consumed at inadequate levels, or vice versa. The NuCal system assigns initial weights to each of the 27 nutrients, and then adjusts these initial weights by the degree to which the nutrient is over or under-consumed relative to recommended intake levels (details in Supplemental File: Methodology and Data Sources in Calculating Nutritional Quality Values for Specific Foods).
4. What to do when a serving of food contains more than 100% of the RDA or comparable intake benchmark for a given nutrient (e.g., cap the maximum score for any one nutrient at 1 or some higher number). NuCal caps the contribution of an individual nutrient at 5 times the applicable RDA or equivalent, a limit that rarely applies when NQI/NuCal values are calculated per serving.
5. Whether and how to include nutritional supplements. NuCal values include additional nutrients in “fortified” food products; values based on just the nutrients in the raw food ingredient can be calculated and are discussed below.
6. How to address fatty acid profiles in animal products and vegetable oils to encourage the shift from heart-unhealthy fats to heart-healthy or neutral ones. NuCal partially addresses fatty acid profiles by including linoleic acid.

Answers to these questions will allow the research community, stakeholders, and the FDA to identify the additional food product testing needed and calculations that will produce a nutritional quality score for a serving of a given food product. The higher the number, the more valuable the food is in meeting a person’s daily nutritional needs. The values can be used to create a continuum with zones ranging from super-foods to those foods delivering little or no nutritional value. A prospective food nutritional quality continuum based on the NuCal metric could be broken into three zones:

- Green Zone—healthiest options (also known as super-foods): NuCal values above 4.
- Yellow Zone—moderately healthy options: NuCal values between 0.5 and 4.
- Red Zone—less healthy options: NuCal values less than 0.5 (also known as junk food, especially when NuCal values fall below 0.1).

Future labeling systems could then highlight where a serving of a given food lies along such a continuum (an example follows). A food product-specific value could also be compared to the average value among competing brands of the same or similar products, or all products in a food group.

### 2.3. Degree of Processing

There are two characteristics of food products that impact nutritional quality and consumer perceptions of healthfulness. One is the degree to which food recipes and manufacturing processes have altered the nutrients in the final product, compared to the nutrients in the raw food ingredients used to make the product. In the NuCal system, there are three food processing metrics. Two are calculated across a set of nutritionally significant nutrients, such as the 27 in NuCal. 

For each nutrient deemed nutritionally significant, the total milligrams of the nutrient in one serving of a food product as sold must be calculated. Then, the quantity of each nutrient in the serving of food from supplements, if any, should be subtracted from the total of that nutrient in the food product. The first metric is the percent share of each nutrient that comes from the raw ingredients in a serving of food (total amount of each nutrient minus amount from supplements, expressed as a percent of total nutrient levels). A second metric reflects the impact of processing, supplements, and fortification on the levels of nutrients in a food product as sold: the percent of each nutrient in the raw ingredients required to manufacture a serving that remain in the final product as sold. These first two metrics can be calculated for each nutrient in the evaluation system and added together in various ways to give a global estimate of the impact of processing and fortification on food nutritional quality.

The second processing and manufacturing characteristic of concern to consumers and the public health community is the number and quantities of ingredients added to the recipe to augment the primary raw food ingredients in some way (preservation, stability, cooking properties, color, mouth feel). A third metric would reflect the number of additives and supplements in the product and the percent of total weight accounted for by such additives.

Gathering these data should be simple. The number of additives can be derived from the ingredient lists that already appear as part of Nutrition Fact panels in the US, as well as somewhere on food packaging in the EU. Food manufacturers would also need to supply the concentrations of each additive in a product’s recipe.

These three simple metrics would provide various ways to quantify the degree to which food processing alters the nutritional content in manufactured food products, and could include the number of such ingredients in a finished food product, the number of ingredients in a manufactured product compared to the ingredients a consumer would typically use at home when making the same or similar food, and/or the combined weight of non-food, added ingredients expressed as a percentage of the grams of raw food ingredients a single serving of the food product.

## 3. Results

Information on the macronutrients in food products is reported in Nutrition Fact panels in the US and similar “Nutrition Information” boxes that appear on EU food products. Data are presented on fat content including total fat and type of fat (saturated, monounsaturated, polyunsaturated, trans fat); cholesterol; sodium; total carbohydrates; fiber; natural sugars and added sugar; and protein. Additional boxes of variable length across food products present the micrograms or milligrams and/or the percent of Daily Value for a generally small number of vitamins, minerals, and other micronutrients in the food product.

Nutrition Facts panels specify serving sizes, as well as the total number of servings in the package. Serving sizes are typically reported in terms of cups, number of pieces, or in grams or ounces (see Figure 1).

**Figure 2:**
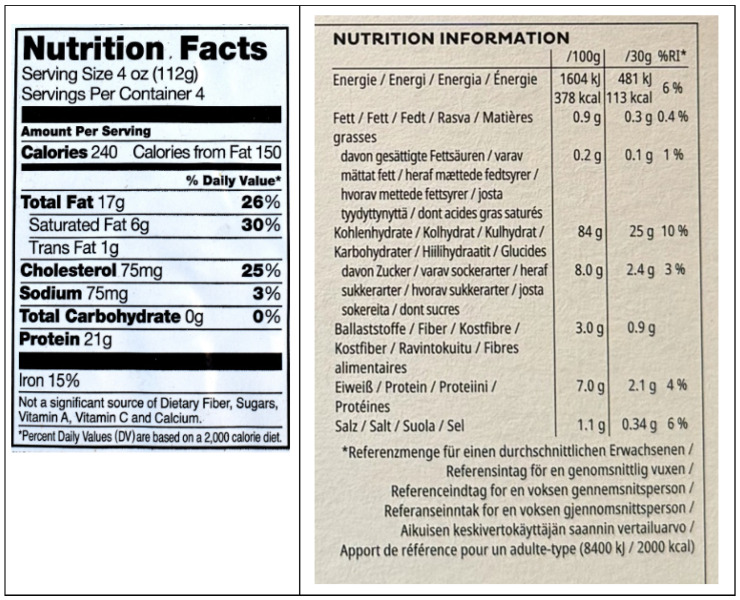
Boxes Providing Information on Macronutrient Composition on Products Sold in the US and EU.

Similar but generally less extensive information is presented on the nutrients in food products sold in the EU (see the labels in Supplemental Tables S1–S18: Front-of-Pack and Back-of-Pack Nutrition and Health-Related Information, Claims, and Messaging on Nine Food Products in the US and the EU). Unlike in the US where all nutrient data are reported per serving, “Nutrition Information” boxes in the EU report nutrient levels per 100 g, and sometimes other quantities such as 30 g or per serving, as shown in Figure 2.

### 3.1. Macronutrient Labeling on Nine Foods Sold in the US and in Europe

The number of nutrient-specific and nutrition health messages on the packaging of the nine food products sold in the US and EU appear in Table 2 and are derived from the 18 Supplemental Tables (two tables per product type in Supplemental Tables S1–S18: Front-of-Pack and Back-of-Pack Nutrition and Health-Related Information, Claims, and Messaging on Nine Food Products in the US and the EU: (1) front-of-pack and (2) back-of-pack, sides, and top/bottom). For the US products, there was a total of 42 messages on front-of-pack labeling, or an average of 4.7 messages per food. On average, there were 3.0 front-of-pack messages containing Content Information, 1.2 messages with Implied Health Benefits, and 0.4 Qualified Health Claims. In back-of-pack and side panel labeling/packaging, there was an average total of 4.1 messages, with Content Information messages accounting for 3.0, or 73%. There were, on average, 1.9 repeat messages on food product packaging, resulting in an average of 6.9 unique messages across the nine US food products.

Among the US products, whole wheat bread and almond milk packaging contained the highest number of unique messages (14, 13). Whole wheat bread contained a total of 22 messages, 8 of which appeared twice for a total of 14 unique messages. There were 11 unique messages on the Cheerios packaging that were heavily weighted toward health benefits. Out of a total of 14 messages on Cheerios packaging, 7 noted Implied Health Benefits and 4 were Qualified Health Claims (including 2 repeated Qualified Health Claims). The packaging of the plant-based ground round (hamburger) contained 8 unique messages, 7 of which entailed Content Information on the FOP. The other five foods contained two to five unique messages, and only one Qualified Health Claim (oatmeal, fiber, and overall health).

The total number and number of unique messages on EU packaging across the nine foods were lower than in the US. In terms of Content Information, there were 19 messages on EU product FOP labeling versus 27 on US FOP labeling. There were 8 FOP Implied Health Benefit messages on EU product packaging, compared to 11 in the US. There were two Qualified Health Claims on the FOP across the nine EU products and four in the case of the US. The same or similar health benefits were associated with nutrient content-related claims on US and EU food packaging.

The biggest difference in the messaging on the US foods in contrast to the EU counterparts occurred in the case of the two plant-based alternatives to animal-based foods: the plant-based ground round and almond milk. Across the two plant-based food alternatives for sale in the US, there were a total of 21 unique messages. There was a total of eight unique messages on the packaging of the comparable EU products, which was only 38% of the US total.

### 3.2. Nutritional Quality Score for Common Foods

The NuCal system nutritional quality scores for specific foods presented below are preliminary and based on a specific set of assumptions, adjustments, and calculations [40]. The US FDA, government agencies in any other country, food companies, or different teams of scientists would surely make different judgements about what nutrients belong in such a calculation, how to quantify nutrient density and compare it to nutrient needs and caloric intake, and whether and how to make adjustments to values based on other considerations (e.g., inadequate versus excess intakes of a given nutrient, the seriousness and/or reversibility of an adverse health outcome brought about by inadequate or imbalanced nutrient intakes).

Table 3 reports NuCal values for 33 widely consumed foods from several food groups, including some multi-ingredient foods. These 33 foods were selected from the Supplemental Table containing 196 foods (see Supplemental File: NuCal and Nutritional Quality Index (NQI) Values for 196 Foods in Three Zones Along the Nutritional Quality Continuum). In Table 3, raw spinach has the highest NuCal value (17.1). A single serving of raw spinach would provide 5.9% of the total nutrient needs, while taking up only 0.35% of an individual’s 2000 calorie daily diet. Oreo cookies had the highest NuCal value in the third, least-healthy red zone (0.48), and is an example of a food that takes up about twice the caloric space relative to the percent of nutrient needs satisfied.

Braised calf liver is by far the most nutritious food per serving in Table 3. A 3 ounce serving provides over 56% of the essential nutrients needed per day in the NuCal system, while accounting for only 8.2% of a 2000 calorie diet (56%/8.2% = 6.8 NuCal value). A medium-sized serving of McDonald’s french fries, however, take up 31% of a person’s caloric allotment, while delivering 16.9% of nutrient needs, for a NuCal value of 0.56. A Big Mac with cheese lands in the red zone with a NuCal value under 0.5 (0.46). A Big Mac with cheese plus a serving of fries would take up 89% of a person’s daily allotment of calories, while delivering almost 44% of the 27 essential nutrients.

Based on caloric data available a few years ago, a Big Mac with cheese, medium fries, and a 12-fluid ounce Coke or Pepsi (i.e., a typical Value Meal) would account for 1929 calories, or all but 71 of a person’s daily 2000 calorie allotment. Such a “Value” Meal also satisfies 44% of daily nutrient needs. But it is not possible to consume the other 56% of daily nutrient needs from foods accounting for only 71 calories. This is why excessive caloric intake from common fast food meals, and not just from McDonalds, is a massive hurdle standing in the way of meaningful progress in improving diet-health outcomes.

The differences in NuCal scores across the three zones of the food nutritional quality continuum are summarized in Table 4. The average food in the healthiest green zone has a NuCal value of 8.1. The 15 foods in the middle, or yellow zone, have an average NuCal value of 1.2, while the average of the 9 foods in the least-health red zone has a NuCal value of 0.2.

Based on the average NuCal values in each zone, food in the green zone delivers almost 33 times more nutrition quality value than the average food in the red zone. Note also that the 9 foods in the green zone have a total NuCal value expressed as a percent of all 33 foods that is about four times higher than the 15 foods in the yellow zone (78.4% versus 19.2%). The 9 foods in the red zone account for just over 2% of the total NuCal values across the 33 foods. This is why efforts to motivate consumers to replace a red zone food choice with a green zone food could make such a substantial contribution to an individual’s health, as well the as attainment of national public health goals.

## 4. Discussion

Most heavily processed, multiple ingredient foods account for a large and growing share of daily caloric intake in many countries and include widely consumed fast foods and beverages that deliver no or very few essential nutrients [43,44], as made clear in Table 4. The food labeling challenge is also enormous. Some 60% of 651 food products marketed for infants and toddlers in the US failed to meet recommended nutrient levels and nearly all products contained one or more prohibited food-health claim, and remarkably, some made up to eleven prohibited claims [12].

Serious deficiencies in micronutrient intakes are surprisingly common in the US and the global food supply. Passarelli et al. report that 4 to 5 billion people on the planet do not consume adequate levels of multiple micronutrients including iodine, Vitamin E, calcium, iron, riboflavin, folate, and Vitamin C, and that women experience greater deficiencies than men [10]. The “What We Eat in America” database reports inadequate intakes for multiple micronutrients across different segments of the US population, with some segments consuming 50% or less than recommended daily intakes [44]. The ratio of omega-6 to omega-3 intakes for the average American exceeds 15:1, and far exceeds the 4:1 or lower ratio that is regarded as heart-health neutral or positive [45,46,47].

For each individual day-to-day, the basic nutrition-related challenge entailed in promoting healthier food choices is conceptually simple: Everyone should consume adequate levels of some two-dozen essential nutrients via food and beverages, and do so while avoiding prolonged, excessive caloric intake.

This is why nutritional quality metrics used to communicate differences across foods via simple front-of-pack label content should ideally be based on overall nutrient content and density relative to caloric space. This will likely prove to be the best option for any front-of-pack nutritional quality labeling scheme that has, as its North Star, the improvement in public health outcomes.

For the foreseeable future, food product labels in the US, EU, and much of the world are likely to contain the following:

- The basic information now presented in macronutrient-focused Nutrition Facts panels or tables;
- Messaging about macronutrient attributes generally or specifically linked to positive health outcomes; and
- Rankings or scores in one or more qualitative systems governing how the food is grown, whether it is bio-engineered, vegan, or organic, and the degree and nature of food processing.

Most US and EU food product labels provide substantial information on macronutrient content. It is widely accepted that front-of-pact nutrition health messaging must be clear, simple, and support healthier food choices. Figure 2, as seen above, is an example of the new FOP graphics the FDA has under consideration that focus on macronutrient composition. Both options in Figure 2 draw upon the data in BOP Nutrition Facts panels. A graphic like those in Figure 2 belongs as part of enhanced FOP nutrition labeling. But the FDA’s two options fall far short of accurately characterizing important differences in the nutritional quality across food products.

Future labeling should be augmented with clear, straightforward graphics, with some conveying the density of nutrients in a serving of food relative to caloric content (including health-linked micronutrients). Many people intuitively understand the need to seek out micronutrient-dense foods and healthier fats, but today’s food labels too often obscure such choices when they should instead clarify and highlight them.

A third graphic is needed that focuses on the degree of processing (discussed below).

Progress on the dietary components of “personalized medicine” [48] will depend to some degree on whether and how future food labeling schemes provide information on aspects of food nutritional quality beyond macronutrient composition, and how food nutritional quality can be customized to reflect different recommended levels of nutrient intake across population cohorts dealing with different diet-related health problems.

Is there room for three graphics and/or crisp narrative messages conveying different aspects of food nutritional quality on the front of food package labeling? As noted in Section 3, FOP labeling on many food packages already contains 10 or more nutrient and nutrition-related messages, many of which are repeated elsewhere on the packaging. The overall impact of FOP labeling on purchase decisions would likely improve if fewer overall messages were delivered that are clearer, actionable, and more fully encompass the overall nutritional quality of the product, as opposed to single nutrients or attributes. In addition, such new labeling must not shy away from conveying the substantial variation in food nutritional quality across types of foods and across competing brands (e.g., potato chips baked in corn oil vs. pita chips baked in canola oil).

**Figure 3:**
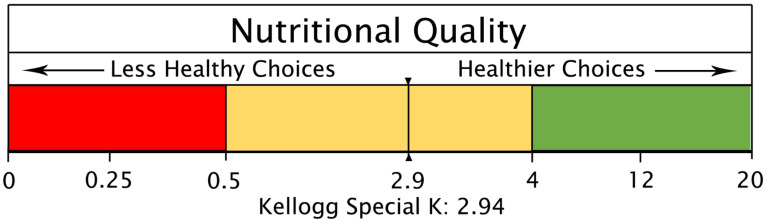
A Front-of-Pack Nutritional Quality Continuum Based on the NuCal Value for Special K Cereal.

Figure 3 below provides an example of how the nutritional quality of a serving of food could be graphically conveyed in the case of the 2.94 NuCal value for Special K cereal (see Supplemental File: NuCal and Nutritional Quality Index (NQI) Values for 196 Foods in Three Zones Along the Nutritional Quality Continuum). This graphic, or variations of it, could include at the bottom “For further information: http//:xx.yy [QR code]”. The link would open up a new tab with a more detailed version of the continuum including details on the source of the Special K NuCal value, how Special K compares to other similar cereals, and information on the percent of nutrients in Special K from the raw food ingredients in comparison to added supplemental nutrients.

### 4.1. Mostly New Challenges in Crafting Simple and Impactful Nutrition Labeling

Analytically, the percent of calories from a serving of a given food is already a part of the Nutrition Facts panel, so this half of the suggested NuCal metric will add no new costs or complexity for companies responsible for generating updated food labeling that adheres to the forthcoming FDA guidance.

Three other labeling challenges are important in terms of motivating and guiding changes in consumer food choices and are discussed below: (1) the degree of “wholeness” in a manufactured food product measured relative to the source of the nutrients in a food product, (2) calculating nutritional quality scores for multi-ingredient foods, and (3) new metrics needed for certain foods that reflect fat quality, and possibly other attributes or issues (e.g., nutrient balances or bioavailability).

The NuCal value for a multi-ingredient food will need to be calculated by quantifying the levels of the essential nutrients in a serving of the food. This process will add modestly to food companies’ costs and burdens, since the levels of essential nutrients can be obtained from analytical labs that are already conducting extensive nutrient-specific testing to support information delivered via Nutrition Facts panels. A relatively small number of samples of multi-ingredient foods would need to be tested annually to confirm the continued accuracy of current nutrient levels in a serving of food.

The quality of the different fats in a serving of food is an important attribute with clear and substantial consequences on public health outcomes [45,46,47,49]. The best available metric to provide an indicator of fat quality is the ratio of omega-6 to omega-3 fatty acids in serving of food [50]. The metric could be based on deviation from a “desirable” or target an omega-6–omega-3 ratio (e.g., a ratio between 1:1 and 4:1 based on published research [51]). Such a metric will be especially valuable in assessing the differences in the nutritional quality of the following:

- Oils derived from olives, canola, corn, soybeans, sunflowers, linseed, or other oilseed crops, including differences across varieties of oilseed crops and production systems [52];
- Grass-fed versus mostly grain-fed cows [53,54], sheep, and chickens [55], and the products derived from them [56,57,58];
- Plant-based versus animal-derived meat, milk and dairy products, and eggs [59,60];
- Wild-caught versus farm-raised fish [61,62];
- Genetically engineered crops and foods derived from animals that are fed GMO feeds or supplements, or perhaps in the future, animals that are genetically altered through the tools of modern biotechnology.

Over time, challenges and new issues will emerge that require reaching agreement on changes in, for example, the nutrients included in calculations, applicable recommended intakes, or how the contribution of each nutrient is weighed in final scores. This process is ongoing in the EU and has recently led to changes in the Nutri-Score system ratings [63,64].

One additional advantage of the recommended approach for nutrition health-related labeling is that nutritional quality scores can be modified in light of the unique nutritional needs of specific population groups (e.g., pregnant women, individuals with GI tract problems) and/or the specific challenges different families and communities face in securing healthy foods. This can be carried out by developing population cohort-relevant adjustments to existing recommended nutrient intake levels, or novel ways to take account of nutrient balance or imbalances in nutritional quality metrics. Nutrient dense fruits and vegetables can be home grown, bought in season, and preserved. Advances in personalized nutrition and food is medicine recommendations to combat chronic diseases and overcome health problems rooted in nutrient deficiencies will be dependent on new ways to quantify the health-promoting potential of different foods and food brands based on their unique distribution of macro- and micronutrient levels.

Table 5 provides an overview of some of the ways nutrition-related guidance and recommendations can be made to consumers via food labels, as well as information portals that can encompass much more detailed discussion of issues, challenges, and possibly useful strategies to accelerate progress toward healthier diets.

Results generated using alternate nutritional quality evaluation systems, and novel metrics like those noted in Table 5, can be made available by the FDA, food companies, and other entities (e.g., the American Dietetic Association) via websites, QR codes, and other means. Such an approach would also allow population subgroup-specific qualified health claims to be sought, approved by the FDA, and communicated via multiple channels. Otherwise, targeted “food is medicine” messages as part of “personalized medicine” would rarely be judged appropriate to appear as a Qualified Health Claim on food packages offered for sale to the general public.

### 4.2. Incorporating the Degree of Processing in Food Labeling

The identification of a robust and useful way to quantify the degree of processing is proving elusive [37,38,39]. Yet the degree of wholeness versus processing is an attribute of food many people care about and pay attention to. Multiple studies report substantial health degradation as consumption of heavily processed food increases (also known as UPF). For example, CVD risk was 7% lower for each 10% increase in plant-sourced, non-UPF intakes, while plant-sourced UPF increased CVD risk and mortality in an UK study [12]. In a study utilizing the NOVA food classification system in the lifelong cohort, a 10% increase in UPF consumption was associated with a 25% increase in risk of type 2 diabetes [21].

Based on the results of several published studies, the NOVA system works reasonably well at the extremes (fresh foods in contrast to heavily processed foods) but struggles to deal with multiple ingredient foods with varying degrees of processing and containing multiple food additives, e.g., [23]. In addition, some UPFs contain substantial nutrients and will score well in most nutrient profiling-based systems, especially if supplemental nutrients in fortified food ingredients and/or added to recipes are counted toward meeting daily nutritional needs.

Novel metrics should be considered in the search for a better way to quantify the degree of processing in a serving of food products. The reasons why consumers and scientists are concerned about the degree of processing should be taken into account in devising better ways to delineate the degree of processing. For many consumers concerned with food nutritional quality, a food brand with fewer chemical additives is generally preferrable, as are products in which most of the nutrients in the raw food ingredients remain in the food product as sold [65].

**Figure 4:**
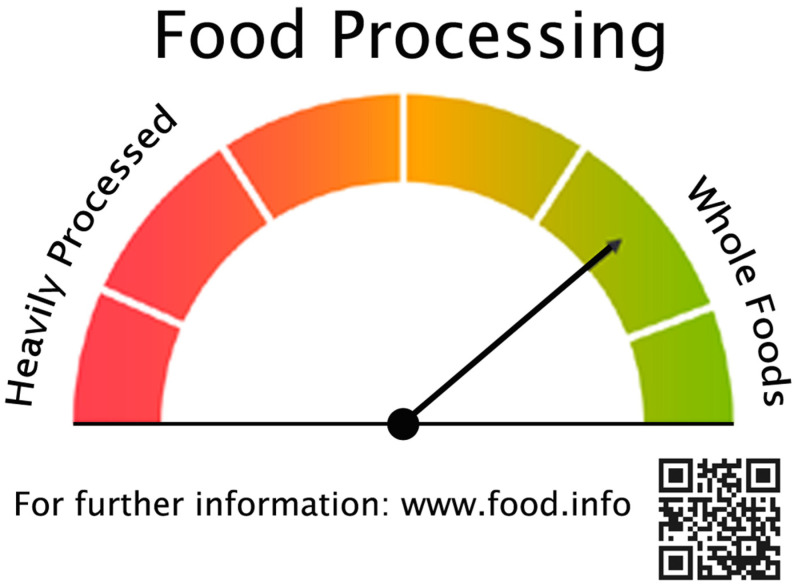
Food Processing Graphic for Placement on Front-of-Pack Labeling.

The factors going into the calculation of degree of processing metrics would vary across food groups and require food companies to conduct some additional tests on their ingredients and finished products as offered for sale. The results could be integrated in a number of ways to produce a metric encompassing the degree of wholeness versus alteration through processing. The values of such a metric could be placed along a continuum or reported as an index. A graphic depicting the degree of processing is presented in Figure 4. As in the case of the nutritional quality continuum graphic in Figure 3, the url and QR code at the bottom of Figure 4 could open a page with additional details on the derivation of the metric, the factors giving rise to the value for a specific food, and additional details such as the percent of nutrients in the product from the raw agricultural ingredients versus food additives, the total number of ingredients in the product, and comparisons to other products or food groups.

**Figure 5:**
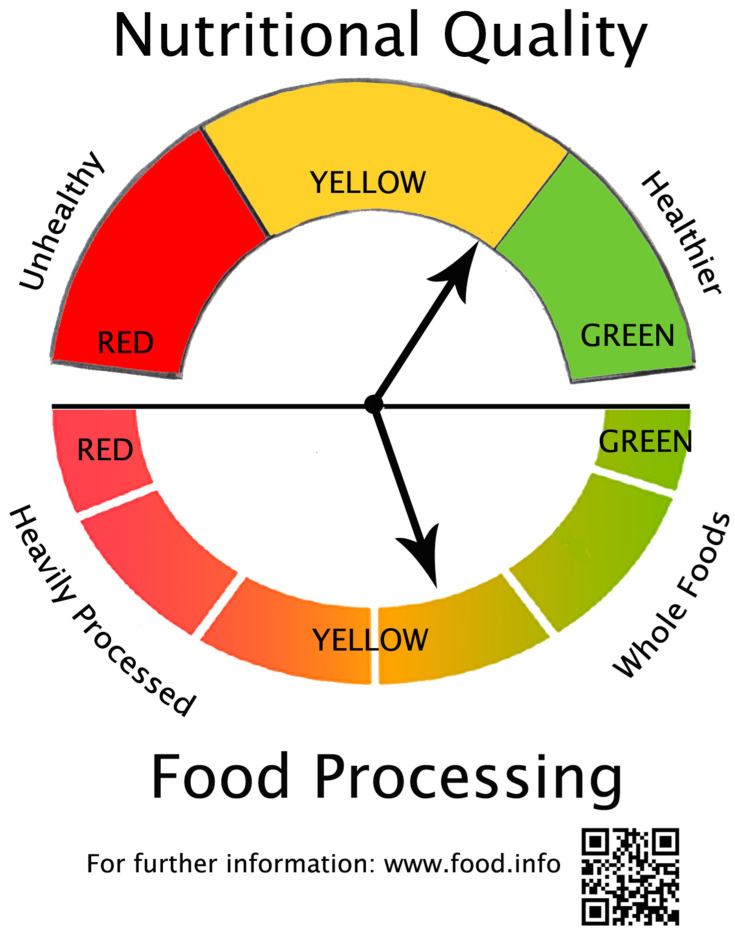
Conveying the Nutritional Quality and Degree of Food Processing in a Food Product in an Integrated Graphic.

The graphic elements in Figure 3 and Figure 4 can be combined in front-of-pack labeling in multiple ways. An integrated option is presented in Figure 5 that combines the nutritional quality and food processing graphics in Figure 3 and Figure 4. As is the case with Figure 3 and Figure 4, the url and QR code at the bottom of Figure 5 could provide access to an explanation of the metrics and how to interpret the information in the graphic, along with more detailed information about data sources and comparisons to other foods. It will take time, education, and consistent messaging from the government, food companies, and health-oriented institutions to help consumers understand what such graphics reflect. But the underlying concepts in such graphics must be grounded in data-driven metrics that accurately convey a food product’s likely contribution, or lack thereof, to a healthy diet.

### 4.3. Motivating Food Companies to Alter Recipes to Make Brand-Name Food Products Healthier

Improved content on front-of-pack and overall packaging that guides consumers toward healthier food choices will incrementally improve health outcomes. But more broad-based progress would be triggered, if, and as new, FDA-approved and mandatory nutrition-focused labeling begins to shift even a few percent of the market share from a brand to its competitors [66,67]. Such a shift would likely lead food manufacturers to review and modify recipes to improve how their brand name products fair in the new labeling scheme, thereby benefitting all consumers.

Like the campaigns deployed to discourage smoking or vaping among teenagers or the use of illegal drugs, government-sponsored public service announcements and related messaging should now be directed toward the need to improve public health via changes in food nutritional quality. A series of 30-s public service announcements informing consumers of the caloric space taken up by popular fast food meals would be an option.

The path ahead for the FDA in putting such a labeling system in place will be fraught with conflicting responses and challenges from food companies, the fast food industry, and commodity groups dedicated to advancing the sales of certain crops and foods. Some of those entities that anticipate benefits as a result of shifting consumer preferences will typically praise newly approved labeling options.

In response, the FDA, or whichever public or private entity decides to act to shift worrisome public health trends through more nutritious foods, will have to withstand substantial criticism and pushback. In the public square, the case will have to be made that there is sufficient information and knowledge today to make progress in what is emerging as this generation’s most pressing public health challenge and opportunity.

## 5. Conclusions

Positive health outcomes will be achieved only if and as consumers eat more nutrient-dense fresh or minimally processed foods, coupled with fewer daily servings of heavily processed foods that deliver little or no nutrients despite prodigious caloric density.

Food package labeling should include clear and actionable information on the levels of macro- and micronutrients in a serving of different types of foods, and across brands within a food group. In addition, better information on the degree of processing will also help many consumers incrementally enhance the nutritional quality of daily dietary choices.

The effectiveness of food package labeling relative to promoting positive public health outcomes will depend on how the various components of nutrient and nutrition health-related labeling work together on food packaging in helping consumers identify better food choices. Focusing on one or a few nutrients or nutrition-health associations will always fall short of what is needed.

The new metrics described above will take time to design, vet, and implement. They will impose some additional, but modest, testing costs on the food industry, especially compared to advertising and marketing expenditures. The FDA will need to resolve a series of computation details in developing and deploying its version of a nutrient profiling system for use in quantifying food nutritional quality. The need for better data and more science will be universally accepted and will never end. Those who call for better information before the FDA or other governments act may, or may not, stymie progress that is clearly attainable now.

Consumer messaging could include a simple rule of thumb—for each serving of food that takes up significant caloric space and delivers modest nutritional value (i.e., red zone “junk food”), be sure to choose one or two servings of green zone super-foods. The possible societal return on investment in developing and deploying new food package labeling could be enormous, especially if and as rigorous and mandatory nutrition labeling motivates food manufacturers to alter recipes to avoid negative nutritional quality or food processing values, and further loss of market share.

Success will depend on a systematic, cohesive, and sustained effort among all stakeholders to work out technical details in ways that support clear and actionable guidance for consumers. Transparent and accurate food product-specific ingredient and nutrient composition data should determine the content of nutrition health labeling. Efforts to soften the message should be resisted in light of the overwhelming need for new food labels that help bring about substantial improvements in food nutritional quality and dietary choices.

## Figures and Tables

**Table 1:** Categories of nutrient and nutrition-health information on food product labels.

	Content	Attributes	Nutrient-Health Outcome
Content Information	“100% Whole Grain”, Vegan, No High Fructose Corn Syrup	Low or no processing, No animal products, more “natural” ingredients	Not specified
Implied Health Benefit	“Good Source”, “Low In”, Nutri-Score value	Linkage of “high” or “low” content claim to some generally recognized positive attribute	Not specified
Qualified Health Claim	Difference in one or more ingredients linked to a specific health outcome	Higher or lower content of an ingredient or nutrient(s) linked to a specific health outcome	Heart healthy; Lowers cholesterol; Improved gut health; Stronger bones

**Table 2:** Significant differences exist in nutrition-related labelling on selected foods in the US and EU.

Food	Brand	Number Front-of-Pack --				Number Back-of-Pack or Other --				Number Repeated	Total Number Unique
		Content Information	Implied Health Benefit	Health Claim	Total	Content Information	Implied Health Benefit	Health Claim	Total		
U.S. Food Brands											
Cheerios	General Mills	2	7	2	11	1	0	2	3	3	11
Oat cereal	Quaker Oats	1	0	1	2	2	0	1	3		5
Pringles	Pringles LLC	2	0	0	2	0	0	0	0	0	2
Whole Wheat Bread	Nature’s Own	9	1	1	11	7	2	2	11	8	14
Plant-based hamburger	Nature’s Promise	6	1	0	7	1	0	0	1	0	8
Whole Milk	Oakhurst	1	1	0	2	2	1	0	3	1	4
Almond Milk	Blue Diamond	4	1	0	5	10	2	0	12	4	13
1000 Island Dressing	Kraft	2	0	0	2	2	0	0	2	1	3
Ketchup	Heinz	0	0	0	0	2	0	0	2	0	2
Average US Products		3.0	1.2	0.4	4.7	3.0	0.6	0.6	4.1	1.9	6.9

**E.U. Food Brands**											
Cheerios	Nestle	8	0	0	8	4	1	0	5	0	13
Oat cereal	Edeka	2	1	0	3	1	1	0	2	1	4
Pringles	Kellogg	1	1	0	2	0	1	0	1	0	3
Whole Wheat Bread	Golden Toast	4	0	2	6	1	0	0	1	1	7
Plant-based hamburger	Edeka	1	2	0	3	2	0	0	2	1	4
Whole Milk	Milsani	1	2	0	3	5	1	0	6	2	7
Almond Milk	Alpro	1	1	0	2	2	1	0	3	1	4
1000 Island Dressing	Kuhne	1	1	0	2	1	1	0	2	1	3
Ketchup	Heinz	0	0	0	0	1	0	0	1	0	1
Average EU Products		2.1	0.9	0.2	3.2	1.9	0.7	0.0	2.6	0.8	5.0

Notes: 1. Nutrient and nutrition-related information on the packaging of EU products has been obtained from the database openfoodfacts (https://world.openfoodfacts.org, accessed on 1 September 2024), and actual packaging on some products sold in Germany. Some of the information and claims on certain products change overtime, and varies from country-to-country in the EU. Thus, our analysis the number of nutrition-related information items on EU food packaging is an approximate snapshot of the nine products in the EU.

**Table 3:** NuCal system values per serving for selected foods in three zones along the nutritional quality continuum.

	% Nutrient Needs Met	Serving Size	Calories	% Daily Caloric Need	NuCal Value
Green Zone (NuCal Value 4 or Greater)					
SPINACH, raw	5.88%	1 cup	6.9	0.35%	17.05
LETTUCE, Romaine	6.12%	1 cup	8.0	0.40%	15.32
BROCCOLI, boiled	12.05%	1/2 cup	27	1.37%	8.83
LIVER, calf, braised	56.20%	3 oz.	164	8.20%	6.85
ALL-BRAN, Kellogg	21.78%	1 ounce	74	3.69%	5.90
CARROT, boiled	7.01%	1/2 cup	27	1.37%	5.13
TOMATO, red	4.05%	1/2 cup	16	0.81%	5.00
STRAWBERRY	10.58%	1 cup	49	2.43%	4.35
GREEN BEANS, boiled	4.66%	1/2 cup	22	1.10%	4.22
Beginning of the Yellow Zone (NuCal Value Between 0.5 and 4)					
ORANGE	7.54%	Medium	62	3.08%	2.45
CHEERIOS, General Mills	12.73%	1 ounce	104	5.21%	2.44
BLUEBERRY	9.89%	1 cup	84	4.22%	2.35
APPLE	5.50%	Medium	67	3.33%	1.65
BREAD, whole wheat	3.72%	1 slice	67	3.33%	1.12
POTATO, boiled, peeled	3.45%	1/2 cup	68	3.39%	1.02
MILK, whole	7.26%	1 cup	149	7.44%	0.98
BANANA	4.93%	Medium	105	5.25%	0.94
OATMEAL, dry	4.87%	1 ounce	108	5.38%	0.90
Pizza Hut cheese pizza	9.62%	1 slice	250	12.48%	0.77
YOGURT, plain, whole	5.49%	1 cup	149	7.47%	0.74
RICE, brown, long grain	3.37%	1 ounce	105	5.25%	0.64
BACON, cooked	4.70%	1 oz.	154	7.70%	0.61
CHEESE, cheddar	3.41%	1 oz.	114	5.71%	0.60
French fries, McDonalds	16.85%	Medium	616	30.78%	0.55
Beginning of the Red Zone (NuCal Value less than 0.5)					
COOKIE, Oreo	3.80%	3 each	159	7.97%	0.48
Chicken pot pie	23.70%	1 pie	1007	50.34%	0.47
Big Mac with cheese	27.09%	1 each	1177	58.83%	0.46
COOKIE, animal crackers	1.96%	1 oz.	127	6.33%	0.31
SYRUP, maple	0.53%	1 Tbsp.	52	2.60%	0.20
BUTTER	0.31%	1 pat	36	1.79%	0.17
GATORADE	0.75%	20 fl. oz.	158	7.92%	0.09
COKE, PEPSI	0.22%	12 fl. oz.	136	6.81%	0.03
SUGAR	0.01%	1 Tbsp.	49	2.44%	0.00

**Table 4:** Average NuCal values per food by zone along the nutritional quality continuum: 33 selected foods.

	Number of Foods	Sum NuCal Values in Zone	Average NuCal Value per Food
Green Zone	9	72.7	8.1
Yellow Zone	15	17.8	1.2
Red Zone	9	2.21	0.25
Totals Three Zones	33	92.6	2.8
Difference Between			
Green and Yellow Zones			6.8
Yellow and Red Zones			4.8
Green and Red Zones			32.9
Percent of Total NuCal Values in Three Zones			
Green Zone		78.4%	
Yellow Zone		19.2%	
Red Zone		2.4%	

**Table 5:** Providing information on smart choices, special needs, and food is medicine as an extension of new food labelling systems ($$ is dollars)..

	Metrics	Applicable to	Foods High/Low in a Recipe	Other Considerations
Smart Choices				
More Nutrition Bang for the Buck	$$ spent per NuCal unit by serving of food	Representative foods in food category (e.g., salty snacks); specific products	$$ spent per NuCal unit by serving of food	Cost of garnishments or accompanying food
Dealing With Nutrient Deficiencies	Nutrient level per serving; Cost per serving	List of common nutrient deficiencies by population cohort	Target nutrients	Avoiding nutrient imbalances; nutrient availability
Strategies for the Winter	NuCal per serving	Perishable and seasonal foods	Target nutrients	Buying in bulk; storage options
Grow Your Own	Space per NuCal unit	Home gardens; in-house options	NuCal value	Family favorites; harvest period
Home Preservation	Safe use time period	Canned, dried, frozen, juices, sauces	NuCal value	Fulfil nutrient needs in winter, off-season
Spices and Ingredients to Boost Nutritional Quality	NuCal per gram/ounce	List of spices, garnishments	NuCal value	How to buy, store, and use high NuCal ingredients

Special Needs				
Pregnancy	Pregnancy adjusted NuCal; TBD	Women pregnant, hoping to become pregnant	Most common nutrient deficiencies	Avoiding nutrient imbalances
Raising Small Children	Infant/child adjusted NuCal values	Families raising children	Most common nutrient deficiencies	Foods kids will eat; healthy snacks
Faith-Based	NuCal Value	Alternate recipes, food choices	Certain ingredients or cooking methods	
Low-Income	$$ spent per NuCal unit by serving of food	Typical $$/NuCal unit for food product	High NuCal per dollar spent	Storage options; compatibility with family favorites
Meeting Nutrient Needs When Fresh Fruit and Vegetables are Scarce or Expensive	$$ spent per NuCal unit by serving of food	Preserved or processed fruit, vegetables, nuts, spices	NuCal vale	Food safety challenges; space for storage

Food as Medicine				
Allergies and Food Sensitivity	NuCal units of alternatives	Target list of common allergies	NuCal units per $$ by serving	
Diabetes and Cardio Vascular Disease	Adjusted NuCal units	Target nutrients with links to CVD	Target nutrients	
Healthy Childhoods	See “Raising Small Children” Above			
GI Tract Ailments				
Combating Inflammation	Anti-inflammatory adjusted NuCal	Foods high in antioxidants	Antioxidant activity	Need for new ways to consume target foods
Sustaining Cognitive Function	Adjusted NuCal units	Foods with phytochemicals linked to brain health	Alternate factors leading to cognitive decline may require different nutrient-related interventions	

## Data Availability

The original contributions presented in the study are included in the article, further inquiries can be directed to the corresponding author.

## Supplementary Materials

The following supporting information can be downloaded at https://www.mdpi.com/article/10.3390/foods13213377/s1, Supplemental Tables S1–S18: Front-of-Pack and Back-of-Pack Nutrition and Health-Related Information, Claims, and Messaging on Nine Food Products in the US and the EU; Supplemental File: Methodology and Data Sources in Calculating Nutritional Quality Values for Specific Foods; Supplemental File: NuCal and Nutritional Quality Index (NQI) Values for 196 Foods in Three Zones Along the Nutritional Quality Continuum.
