# Supplementary material for: Enhanced Labeling to Promote Consumption of Nutrient Dense Foods and Healthier Diets
Source: Foods. 2024 Oct 24;13(21):3377. doi: 10.3390/foods13213377 (PMC11544848; doi:10.3390/foods13213377)
Supplement: Supplementary file 1 [file foods-13-03377-s001.zip › Suppl File_NuCal_Method_10-18-24.pdf]

Supplemental File: Methodology and Data Sources in Calculating Nutritional Quality Values for Specific Foods

The NuCal metric and labeling system combines the nutrient density in a serving of food relative to the required daily intakes of each specific nutrient, with the caloric density of a serving of food (i.e. the familiar number of calories per serving divided by 2000 calories in a typical daily diet).

The core components of the nutrient profiling system used in calculating NuCal values are explained herein. This system was originally developed by Dr. Donald R. Davis and Dr. Charles Benbrook to support food nutritional-quality research undertaken by The Organic Center.

The "Nutritional Quality Index" (NQI) provides a broad-based measure of the nutritional benefits of individual foods, meals, and daily diets. It is designed to analyze food choices in ways that will support attainment of public-health outcomes. It is based on nutrient levels in a single serving of food relative to the recommended daily intake of each nutrient.

RDAs and comparable intake thresholds are intended to meet the requirements of nearly all normal, healthy persons, including those with above-average needs. "Adequate Intakes," or AIs, are preliminary recommendations for nutrients for which there are insufficient data, or lack of consensus, on the basis to establish an RDA.

The NQI for a given food is based on its content of 27 nutrients, encompassing minerals, vitamins, antioxidants, protein, fatty acids, and fiber. Individual nutrients can be weighted according to their abundance or shortage in American diets, placing greater emphasis on those nutrients typically falling short in contemporary American diets.

Special-purpose NQIs can also be calculated for individuals or population groups with nutritional needs that differ from the typical adult consumer (e.g. caloric intake, need for added fiber, Vitamin D). The current version of the NCI is based on the nutritional needs of a woman ages 19 to 30.

A. Parameters in the NQI and Data Sources

Per-serving NQIs are especially useful for comparing the broad nutrient contribution of different categories of food (grains versus dairy versus fruits), as well as between two foods within the same category (e.g., white vs. brown rice, whole vs. skim milk, potato chips versus pita chips, organic vs. conventional blueberries). Two directionally correct strategies to improve diets are to choose: (1) more servings per day from categories of food with relative high NQI scores (fresh fruits and vegetables), and (2) between similar foods based on their NQI values per serving.

Because the serving sizes and density of different foods can vary widely, NQIs ***per serving*** are often not very comparable. For example, a half-cup serving of carrots contains 27 calories compared to only 8 calories in a one-cup serving of lettuce. This calorie difference contributes to a large difference in NQI per serving, 0.07 for carrots and 0.02 for lettuce. But on an equal-calorie basis, the NQIs are the same for carrot and lettuce, 0.26 per 100 calories. Similar issues arise in comparing NQI values per equal weights of different foods (e.g. 100 grams), an approach markedly biased against beverages, as well as fresh fruits and vegetables or other foods high in water content.

The three different ways to calculate and express NQI values—per serving, per 100 calories, and per 100 grams—add different insights and can play a role in different kinds of analytical exercises and comparison. Any one of the three can be calculated from either of the other two.

<u>Nutrients Included</u> The NQI value for a single food is based on its content of 27 nutrients —eleven vitamins, eight minerals, protein, fiber, choline, linoleic acid, linolenic acid, lycopene, lutein + zeaxanthin, and total ORAC. The 27 items include diverse "phytochemicals" that are associated with a range of positive public health outcomes, even though some are not considered essential nutrients by the Food and Drug Administration (FDA) and lack official RDAs or AIs.

Based on the importance of these nutrients to the health and quality of life for most Americans, they are included within the NQI algorithm using a two-step process. First, the following initial nutrient shares, or weights, are established that add up to 100%:

- Eleven vitamins – 26% (2.4% per vitamin)
- Eight minerals – 19% (2.4% per mineral)
- Protein – 16%
- Fiber – 10%
- Antioxidant activity as measured by total ORAC – 8%
- Lutein + zeaxanthin – 5%
- Linoleic acid – 5%
- Linolenic acid – 5%
- Lycopene – 3%
- Choline – 2.4%

The 11 vitamins include A, D, E, K, B_6, B_{12}, C, folate, thiamin (B_1), riboflavin (B_2), and niacin (B_3).

The eight minerals include calcium (Ca), potassium (K), magnesium (Mg), phosphorus (P), copper (Cu), iron (Fe), selenium (Se), and zinc (Zn).

Initial shares of 2.4% for vitamins and minerals are then allotted equally. Protein, fiber, and ORAC have larger shares, because they consist of multiple substances, e.g., the nine essential amino acids in protein. Linoleic acid and linolenic acid are precursors for other

fatty acids. Lutein, zeaxanthin, and lycopene are important antioxidants not significantly measured by the ORAC assay.

These weightings are subjective estimates with an emphasis on biologically active compounds found especially in fresh, whole foods. Heightened importance is placed on phytochemicals because of:

- The many suspected health-promoting properties of antioxidants in food,
- The emphasis placed on increasing intakes of fresh, whole fruits, vegetables, grains, and nuts in the most recent *Dietary Guidelines* from the USDA,
- The general consensus that antioxidant intakes need to at least double across the population to optimally combat the damage triggered by reactive oxygen and nitrogen species (so-called "free radicals"), and
- The growing evidence that phytochemical intake is critical in slowing the aging process and the progression of many adult-onset diseases.

Weights assigned to specific nutrients can be changed as new information becomes available, or when the NQI is applied to a sub-population with special nutritional needs.

The second step in establishing final weights for each of the 27 nutrients is driven by their relative abundance or deficiency in typical American diets compared to recommended intakes. In this step for a given population group, the share assigned to each nutrient in Step One is multiplied by the inverse of the population group's average intake compared to the applicable RDA or AI. This method strives to take into account the relative need for increased/decreased intakes of specific nutrients based on typical dietary patterns in a given population cohort.

For example, if the average intake of a given nutrient is only half of the applicable RDA or AI, we increase that nutrient's weight by 1/0.5 = 2. This ratio is an estimate of the "degree of deficiency" of the nutrient. If the average intake for a nutrient is twice the RDA, that nutrient's weight is reduced by 0.5 (1/2 = 0.5). There is no adjustment if the average intake equals the RDA (deficiency = 1).

Final "index weights" for the 27 nutrients in the NQI are these deficiency-adjusted shares, normalized so that the total index weight across all 27 nutrients = 1 for each population group. Details are shown in the Appendix below.

The 27 final index weights can change in two situations: (1) adjustments in the initial shares for each nutrient (e.g., protein's 16% share), and more commonly, (2) changes in the recommended intakes or "deficiency" factors (average intakes/recommended intakes).

In general, deficiency factors will vary for each population group in which average intakes and/or recommended intakes vary from cohort to cohort. In either situation, the total of 27 index weights is renormalized to 1, so that NQIs remain comparable among diverse population groups, and the one-day NQI remains 1 for someone who consumes exactly the RDA or AI for each of the 27 nutrients.

Nutrient Needs Nearly all of the food-specific nutrient content data comes from the U.S. Department of Agriculture's "National Nutrient Database for Standard Reference" (USDA-ARS, 2010). As needed and possible, data from other sources is utilized (e.g. most antioxidant levels are from a USDA ORAC [Oxygen Radical Absorbance Capacity] dataset). For nutrients with a recommended daily intake level, the system depends upon the Recommended Dietary Allowances (RDAs) of the Food and Nutrition Board of the Institute of Medicine, or the Board's estimated "Adequate Intakes" (AIs) for a few nutrients lacking a formal RDA.

There are no official government RDAs/AIs, or DVs for the phytochemicals lutein, zeaxanthin, and lycopene, nor for measures of antioxidant capacity such as ORAC. To include these items in the NQI, AIs were estimated based on intakes in recommended diets and/or emerging evidence on the health benefits of these substances, including epidemiological studies exploring the impacts of various intake levels on human health.

Lutein and zeaxanthin are important, but little-known, carotenoids with significant antioxidant capacity. Both concentrate in our eyes and protect them from damage by light. Foods such as spinach, other greens, and yellow corn are good sources of these yellow-orange pigments. They are chemically so similar to each other that many analytical methods do not distinguish between them. For this reason, the levels of lutein and zeaxanthin in foods are usually reported together as a sum.

Based on published research, an adult "AI" for lutein + zeaxanthin has been set at 2000 micrograms per day. Typical daily intakes in the U.S. range from less than 1000 micrograms to over 5000 micrograms. The popular multi-vitamin Centrum Silver contains 250 micrograms of lutein + zeaxanthin, while some stand-alone lutein + zeaxanthin supplements contain as much as 6000 micrograms.

Lycopene is another antioxidant and carotenoid pigment linked to positive health outcomes. It accumulates in the liver and other organs, and is being studied for possible roles in helping prevent prostate, lung and other cancers. Tomatoes, and especially cooked tomato products, are the major sources of lycopene for most Americans. The few other significant sources of lycopene are watermelon, papaya, red-fleshed guava, and pink grapefruit. Our "AI" of 10,000 micrograms per day is based on average U.S. intakes ranging from 5000 to over 15,000 micrograms per day in various population cohorts. A cup of stewed tomatoes contains 10,000 micrograms of lycopene, and a cup of watermelon, 7000 micrograms. The Centrum Silver supplement contains 300 micrograms.

ORAC is a leading measure of antioxidant capacity of foods, now reported by USDA for several hundred foods, mainly fruits and vegetables (ORAC, 2010). A few values for grains, beans, nuts, milk, spices, and chocolate also show substantial antioxidant activity in the ORAC assay. More complete ORAC data is needed for these foods. USDA researchers have estimated a U.S. average daily consumption of 5700 ORAC units from fruits and vegetables alone, corresponding to about 2.5 servings per day of fruits and vegetables. They have noted that the recommended 8-9 servings per day of fruits and

vegetables would supply about 20,000 ORAC units. For NQI calculations, a provisional adult "AI" of 20,000 ORAC units is used, with a U.S. average consumption of 10,000 ORAC units, including poorly known contributions from grains, beans, nuts, milk, spices, and chocolate.

Eating high-ORAC foods like apples, blueberries, and artichokes raises the antioxidant power of human blood. Animal studies suggest that high-ORAC foods combat oxidative stress and can slow aging processes that can impair memory and learning ability. One cup of raw blueberries provides 6900 ORAC units, and organic blueberries provide even more (about 50% more according to a 2008 report by Wang, et al.). Several common fruits and vegetables provide 1500 to 7000 ORAC units per serving, especially dark, intensely colored ones like red grapes, red apples, strawberries, asparagus, and broccoli.

Nutrient Levels in Foods The USDA's online nutrient database is considered the most reliable, extensive, and up-to-date source of nutrient content data for foods consumed in the U.S. For most common foods, USDA purchases food products from various outlets in order to develop a nationally representative sample.

Nutrient Intake Levels Data on dietary intakes comes from large, nationally representative surveys conducted by the U.S. Department of Agriculture (USDA) and the U.S. Department of Health and Human Services (USDA and HHS, 2007). Average nutrient intakes in the U.S. from the "Usual Intakes from Food" are incorporated in the NQI. These average intakes are conveniently shown for the same age and population groups that are used for reporting RDAs and AIs.

For intakes of lycopene and lutein + zeaxanthin, average intakes from the NHANES for 2007-2008 were incorporated in the NQI, using its age and population groups that most closely match the RDAs in the NQI system (e.g., females age 20 to 29 instead of age 19 to 30 for the RDAs).

B. Estimating the Nutritional Quality Index of a Single Food

When applying the NQI to the nutritional needs of a given person, two issues arise in determining the appropriate weighting factors to use across the 27-nutrients. The first issue is whether the RDAs and "initial shares" described above may differ for a specific individual, compared to population averages, because of some health condition, medication the person is taking, or perhaps differences in genetics or digestive system health.

One way to accommodate such special cases is to adjust the initial shares assigned to specific nutrients, while another option would be to adjust the RDAs. The ability to take into account variability in the health status and nutrient needs of individuals is an advantage of the NQI and similar nutrient profiling systems that could help advance "personalized medicine" drawing upon "food as medicine".

The second issue is that an individual's average daily intakes of specific nutrients might differ markedly from their population cohort averages. In such a case, using the cohort average intakes to calculate the "deficiency" weights described above (based on the adequacy/inadequacy of intakes relative to the RDAs/AIs) will skew NQI values.

Dealing with Exceptionally High Levels of Nutrients in Some Foods A few foods have extraordinarily large amounts of certain nutrients, for example, vitamin K in kale. To prevent any one nutrient from contributing excessively to a food's NQI value, a maximum nutrient content cutoff of five RDAs per 100 calories of food. Such cutoff values are particularly needed when comparing NQI values across foods per some set number of calories. This is because some fruits and vegetables have high levels of certain nutrients coupled with relatively low caloric content.

This 5-RDA cutoff was chosen to identify the few nutrient-food combinations in which extremely disproportionate levels of certain nutrients are present. It affected almost exclusively vitamin K and lutein + zeaxanthin in green, leafy vegetables. It also occurred with three nutrients in liver (copper and vitamins A and B_{12}). A method to truncate extreme contributions to NQI values for specific nutrients is also justified biologically, because multi-RDA amounts in a single serving may not be well absorbed nor enhance health.

Spinach is a good example. A one-cup serving of raw spinach (only 7 calories) contains about 1.6 RDAs each of vitamin K and lutein + zeaxanthin (for women age 19 to 30). A person would need to consume over 14 cups of spinach to ingest 100 calories, a quantity of spinach delivering almost 23 times the RDAs for vitamin K and lutein + zeaxanthin.

Nutritional Quality Indexes for A Few Foods The table below reports the NQI values for 10 vegetables.

	Nutritional Quality Index				
**Top 10 U.S. Vegetables by Retail Weight**	**Per 100 grams**	**Per 100 Calories**	**Per Serving**	**Serving Size**	**Serving Calories**
Potato, boiled in skin & peeled	0.044	0.051	0.034	1/2 cup	68
Onion, boiled	0.030	0.067	0.031	1/2 cup	46
Lettuce, iceberg	0.037	0.262	0.021	1 cup	8
Tomato	0.045	0.250	0.041	1/2 cup	16
Lettuce, Romaine	0.130	0.766	0.061	1 cup	8
Bell pepper, green	0.061	0.303	0.046	1/2 cup	15
Corn, yellow	0.074	0.077	0.056	1/2 cup	72
Carrot, boiled	0.090	0.257	0.070	1/2 cup	27
Cabbage, boiled	0.066	0.286	0.049	1/2 cup	17
Cucumber, with skin	0.018	0.119	0.009	1/2 cup	8
Average	0.059	0.244	0.042		29

Of all the food groups, vegetables pack the most nutrients into relatively few calories, shown here by their exceptionally high average NQI of 0.24 per 100 calories. Typical servings of vegetables have a NQI of 0.03 to 0.05, at the expense of only 10 to 50 calories. Unfortunately, less than one in three Americans meets the minimal goal of three servings of vegetables per day.

Nutrients that contribute the most to the NQIs for vegetables include vitamin K, lutein + zeaxanthin (both mainly in green vegetables), fiber, vitamins A and C, and ORAC. Vegetables also contribute many other vitamins and minerals.

	Nutritional Quality Index				
**Top 10 U.S. Fruits by Retail Weight**	**Per 100 grams**	**Per 100 Calories**	**Per Serving**	**Serving Size**	**Serving Calories**
Banana	0.042	0.047	0.049	Medium	105
Apple	0.043	0.083	0.055	Medium	67
Watermelon	0.036	0.119	0.054	1 cup	46
Cantaloupe	0.036	0.105	0.056	1 cup	53
Orange	0.058	0.122	0.075	Medium	62
Grape	0.035	0.050	0.052	1 cup	104
Strawberry	0.070	0.218	0.106	1 cup	49
Pineapple	0.033	0.067	0.055	1 cup	83
Peach	0.039	0.099	0.058	Medium	59
Avocado	0.111	0.069	0.083	1/2 cup	120
Average	0.050	0.098	0.064		75

Fruits have a smaller average NQI of 0.10 per 100 calories, generally because of the higher carbohydrate levels in many fruits. Typical servings contribute 0.05 to 0.10 NQI units in 50 to 100 calories, with strawberries as a clear nutrient-density standout at 0.22. In fruits, the largest contributors to NQI are ORAC, fiber, and vitamin C. The carotenoids lutein, zeaxanthin, and lycopene contribute heavily to NQI values for a few fruits, especially lycopene in watermelon.

	Nutritional Quality Index				
**Whole Grains**	**Per 100 grams**	**Per 100 Calories**	**Per Serving**	**Serving Size**	**Serving Calories**
Whole wheat flour	0.191	0.056	0.054	1 ounce	97
Brown rice, raw	0.119	0.032	0.034	1 ounce	105
Corn meal, whole	0.170	0.047	0.048	1 ounce	103
Oatmeal, dry	0.171	0.045	0.049	1 ounce	108
Rye flour, dark (whole)	0.285	0.088	0.081	1 ounce	92
Wild rice, raw	0.171	0.048	0.048	1 ounce	101
Barley (whole), raw	0.221	0.062	0.063	1 ounce	101
Triticale flour, whole	0.196	0.058	0.056	1 ounce	96
Amaranth grain, raw	0.181	0.049	0.051	1 ounce	105
Kamut grain, raw	0.190	0.056	0.054	1 ounce	96
Average	0.189	0.054	0.054		100

Whole grains include the nutrient- and fiber-rich bran and germ of the seed kernel, both of which are removed from white flour and white rice. The germ is removed from most corn meal. Whole grains contribute about 0.05 NQI units per 100 calories, less than vegetables and fruits, but valuable as low-cost sources of protein, fiber and many other nutrients. Consumption of whole grains is increasing, but is still far short of recommended intakes (at least half of total grain intake should be whole grains). Whole

grains are believed to reduce risks for heart disease, stroke, obesity, cancer, type-2 diabetes, and kidney stones. The fiber in grains also helps prevent constipation.

The largest contributors to grain NQIs are fiber, protein, and ORAC. Lutein + zeaxanthin stands out in corn meal.

	Nutritional Quality Index				
**Whole Grain Products**	**Per 100 grams**	**Per 100 Calories**	**Per Serving**	**Serving Size**	**Serving Calories**
Bread, whole wheat	0.138	0.056	0.037	1 slice	67
Bread, 7-grain (whole)	0.141	0.053	0.037	1 slice	69
Shredded Wheat cereal	0.170	0.050	0.048	1 ounce	96
Rice cake, brown rice	0.110	0.028	0.030	2 cakes	104
Wheaties cereal	0.488	0.141	0.139	1 ounce	99
Cheerios cereal	0.448	0.122	0.127	1 ounce	104
All-Bran cereal	0.767	0.295	0.218	1 ounce	74
Average	0.323	0.107	0.091		87

Some breads and cereals such as Shredded Wheat are made with whole wheat, so their NQIs per 100 calories are similar to whole wheat. Unlike Shredded Wheat, Wheaties, Cheerios and All-Bran cereals also contain added nutrients accounting for 10% to 100% of the RDAs in a single serving. Such fortification doubles or triples their NQIs compared to the grain ingredients alone.

The major contributors to these NQIs are fiber, protein and the added vitamins and minerals. All-Bran cereal's extraordinary nutrient density comes partly from the high fiber content of bran. However, because bran lacks the germ and starchy part of wheat kernels, it is not fully whole grain, and the cereal contains significant added sugar.

	Nutritional Quality Index				
**Refined Grain Breads**	**Per 100 grams**	**Per 100 Calories**	**Per Serving**	**Serving Size**	**Serving Calories**
Bread, wheat, enriched	0.110	0.041	0.027	1 slice	67
Bread, white, enriched	0.094	0.035	0.023	1 slice	67
Bread, rye	0.111	0.043	0.022	1 slice	52
Bread, oatmeal	0.099	0.037	0.027	1 slice	73
Bread, French	0.096	0.033	0.024	1 slice	72
Cornbread	0.075	0.024	0.021	1 ounce	89
Average	0.098	0.036	0.024		70

Note that the NQI per 100 grams for refined grain breads is only about 0.10 even after being "enriched," compared to about 0.15 for whole grain breads. White flour is the leading ingredient in most breads, including some with names that may suggest otherwise, such as Bran and Wheat Bread, Wheat Bread, most Multigrain Breads, and Oatmeal Bread. Only 100% Whole Wheat Bread is certain to list no white flour as an ingredient. White flour can be confusingly labeled as "flour," "wheat flour," or "unbleached flour."

The white flours used in all these breads are "enriched," meaning they contain added amounts of ~five nutrients that modestly boost their NQIs (typically thiamin, riboflavin,

niacin, iron, and folate). Still, their NQIs are less than the values for whole grain breads, reflecting their losses of fiber, magnesium, potassium, vitamin B_6, and other nutrients that are not added.

USDA's *Dietary Guidelines* recommend reduced consumption of refined grains. The leading contributors to the depleted NQIs shown here are protein, fiber and ORAC, including for white bread.

	Nutritional Quality Index				
**Refined Grain Cereals**	**Per 100 grams**	**Per 100 Calories**	**Per Serving**	**Serving Size**	**Serving Calories**
Corn Flakes cereal	0.323	0.089	0.092	1 ounce	103
Corn Pops cereal	0.254	0.065	0.072	1 ounce	110
Rice Krispies cereal	0.333	0.086	0.095	1 ounce	110
Special K cereal	0.557	0.147	0.158	1 ounce	108
Froot Loops cereal	0.288	0.077	0.082	1 ounce	106
Frosted Cheerios cereal	0.329	0.087	0.094	1 ounce	107
Average	0.348	0.092	0.099		107

Refined grain cereals generally lack bran and germ, and most of them contain 20% to 40% added sugar. However, because of substantial fortification with some nutrients, their average NQI of about 0.09 per 100 calories looks much better than refined grain breads (0.02 to 0.04 per 100 calories), and better even than whole grains (about 0.05 per 100 calories). The added nutrients dominate these NQIs. Unfortunately, the added nutrients do not make up for refining losses such as fiber, magnesium, potassium, and ORAC. Despite the added nutrients that enhance NQIs, the USDA recommends reducing consumption of refined grain products and choosing whole grain cereals with minimal added sugar.

	Nutritional Quality Index				
**Dairy Foods**	**Per 100 grams**	**Per 100 Calories**	**Per Serving**	**Serving Size**	**Serving Calories**
Milk, whole (3.3% fat)	0.030	0.049	0.073	1 cup	149
Milk, 2% fat	0.028	0.056	0.068	1 cup	122
Milk, 1% fat	0.028	0.067	0.068	1 cup	102
Milk, nonfat	0.028	0.083	0.069	1 cup	83
Milk, soy (fortified)	0.036	0.084	0.088	1 cup	104
Cheese, cottage, 4.5% fat	0.038	0.039	0.044	1/2 cup	111
Cheese, cottage, 1% fat	0.038	0.053	0.043	1/2 cup	81
Cheese, American	0.110	0.029	0.031	1 oz.	106
Cheese, Cheddar	0.120	0.030	0.034	1 oz.	114
EGG, raw	0.111	0.078	0.056	1 large	72
Average	0.057	0.057	0.057		105

Milk is an important source of high-quality protein, calcium and added vitamin D in most American diets. As the fat and its calories are progressively removed from whole milk to make reduced-fat milk and dairy products, the NQI per 100 calories increases, because few nutrients are removed with the fat, and the calories decline substantially. However, the NQI per serving declines slightly, because of the loss of linolenic acid, an omega-3

fat. Other omega-3 fats, conjugated linoleic acids, and other beneficial substances not included in the NQI, are also removed, which is one of the reasons why whole milk is gaining favor from the nutrition community. The broad nutrient density of eggs is well above most other dairy products.

Soymilk has higher amounts of some nutrients than cow's milk, and is often fortified with calcium and vitamins A, B_{12}, and D, to levels similar to cow's milk. During cheese making, fat and calories are concentrated, but some nutrients in milk are lost in the removed whey. These changes and the lack of added vitamin D decrease the NQIs per 100 calories for cheeses, especially for solid cheeses such as American and Cheddar.

On average, American adults consume about half of the USDA-recommended 3 cups per day of reduced-fat milk and milk products. These products (but not fatty cheeses) are recommended for bone health and reduced risk of heart disease, type-2 diabetes, and hypertension (USDA's *Dietary Guidelines for Americans*). Protein, vitamin D, and calcium are the leading contributors to most of these TOC-NQIs. In eggs, choline and lutein + zeaxanthin stand out.

	Nutritional Quality Index				
**Meats and Sea Foods**	**Per 100 grams**	**Per 100 Calories**	**Per Serving**	**Serving Size**	**Serving Calories**
Ground beef, cooked (15% fat)	0.109	0.047	0.092	3 oz.	198
Calf liver, braised	0.659	0.343	0.562	3 oz.	164
Pork loin, roasted	0.119	0.048	0.101	3 oz.	211
Pork spareribs, roasted	0.113	0.047	0.096	3 oz.	203
Chicken, whole, roasted	0.103	0.043	0.087	3 oz.	204
Chicken breast, fast food	0.093	0.031	0.079	3 oz.	258
Salmon, Atlantic, baked	0.196	0.095	0.167	3 oz.	176
Tuna, light, canned in water	0.127	0.109	0.108	3 oz.	99
Catfish, baked	0.080	0.056	0.068	3 oz.	123
Shrimp, boiled	0.104	0.088	0.089	3 oz.	101
Average	0.170	0.091	0.145		174

Meats and sea foods supply high quality protein and many other nutrients. Besides protein, leading contributions to NQI come from vitamin B_{12} in beef, calf liver, and sea foods, thiamin in pork, linoleic acid and niacin in chicken, vitamin D in salmon and tuna, and choline in catfish and shrimp. All these foods are important sources of many other nutrients, including linolenic acid and other omega-3 fatty acids in sea foods and chicken. Calf liver is an extraordinarily rich and diverse food.

According to the USDA, some Americans need to increase their intake of protein foods, while most get more than its recommendation, especially of beef (*Dietary Guidelines for Americans*). Based on evidence that the omega-3 fatty acids EPA and DHA help prevent heart disease and death from heart disease, USDA recommends that Americans increase their consumption of a variety of sea foods to 8 ounces per week, especially those low in mercury.

Sweets and Added Fats	Nutritional Quality Index Per 100 grams	Per 100 Calories	Per Serving	Serving Size	Serving Calories
Sugar	0.001	0.000	0.000	1 Tbsp.	49
Honey	0.005	0.002	0.001	1 Tbsp.	64
Oil, soybean	0.431	0.049	0.059	1 Tbsp.	120
Oil, olive	0.110	0.012	0.015	1 Tbsp.	119
Butter	0.061	0.009	0.003	1 pat	36
Margarine (stick)	0.137	0.022	0.007	1 pat	31
Coke and Pepsi Colas	0.001	0.002	0.002	12 fl. oz.	136
Gatorade, fruit-flavored	0.001	0.005	0.007	20 fl. oz.	158
Cookie, Oreo	0.112	0.024	0.038	3 each	159
Cookie, animal crackers	0.069	0.015	0.020	1 oz.	127
Caramel candies	0.038	0.010	0.011	1 oz.	108
Chocolate chips, semisweet	0.214	0.045	0.061	1 oz.	136
Average	0.098	0.016	0.019		104

Refined sugars, added fats, and foods high in these ingredients rank mostly at the bottom of the nutrient density scale, with NQIs of 0.00 to 0.02 per 100 calories. In its *Dietary Guidelines for Americans,* USDA recommends minimizing most of these foods with low nutrient density. However, it distinguishes between sugars and solid fats on one hand (including butter and margarine), and oils on the other hand, because oils contain some valuable nutrients. The relatively high NQI for soy oil, for example (0.049 per 100 calories), comes mainly from linoleic and linolenic acids, with only 0.008 per 100 calories from other nutrients.

Olive oil contains relatively little of these nutrients, as it contains mostly monounsaturated fats (not included in NQI). Most of the NQI for chocolate chips and some of the NQI for Oreo cookies comes from ORAC, with contributions also from other nutrients in cocoa beans. USDA notes that soft drinks and sports drinks like Gatorade are leading sources of refined sugars in American diets, and many Americans drink too much of them. The partial hydrogenation used to make stick margarines destroys some linoleic and linolenic acids and has the further disadvantage of producing unhealthy *trans* fatty acids.

NQIs can also be calculated for food mixtures such as sandwiches, other entrees, recipes, complete meals and even complex diets. Potentially two different methods can be used.

Method 1

For many relatively common and simple food mixtures, the USDA reports the nutrient contents of the mixtures in the same way that it does for single foods, and the NQI calculation proceeds in the same way as well. Some examples are shown below.

	Nutritional Quality Index				
Fast Foods & Other Mixtures	Per 100 grams	Per 100 Calories	Per Serving	Serving Size	Serving Calories
Big Mac with cheese	0.130	0.023	0.271	1 each	1177
French fries, McDonalds	0.148	0.027	0.168	Medium	616
Shrimp, breaded, fast food	0.094	0.021	0.154	6-8 shrimp	745
Onion rings, breaded, fast food	0.031	0.011	0.025	8-9 rings	229
Pizza Hut cheese pizza	0.100	0.039	0.096	1 slice	250
Chicken pot pie	0.109	0.024	0.237	1 pie	1007
Ice cream, vanilla	0.026	0.019	0.017	1/2 cup	90
Ham & cheese sandwich	0.094	0.027	0.138	1 each	514
Burrito, bean & cheese	0.107	0.028	0.198	2 each	703
Fried chicken, fast food	0.152	0.031	0.248	Breast & wing	805
Average	0.099	0.025	0.155		614

Notice in these examples that the NQIs per 100 calories are low compared to most whole foods, because of the substantial additions of frying fat, refined sugar, and white flour. For example the NQIs per 100 calories are 0.023 for a Big Mac, compared to 0.047 for ground beef, 0.027 for French fries compared to 0.051 for boiled potato, 0.021 for fried and breaded shrimp compared to 0.088 for boiled shrimp, and 0.011 for fried and breaded onion rings compared to 0.061 for boiled onions.

Method 2

For food mixtures, entrees, meals and diets that are not reported by USDA, the calculation of NQI is more complex. It requires adding up the weights, calories, and 27 nutrients from each of the individual foods in the mixtures. The summed values are then used in the same way as for single foods. Some commercially available recipe- and diet-analysis programs can perform the needed summations. NutriCircles software (Strickland Computer Consulting, 2011) has been used in the NQI calculations discussed herein. It contains nearly 4000 foods and mixtures from the USDA's current Nutrient Database for Standard Reference.

Alternatively, the nutrient content of food mixtures can be measured by a qualified laboratory, a process that would cost over $2000 per sample. One analytical laboratory charges $600 to $800 for just the mandatory nutrients on a Nutrition Facts food label, which does not include 20 nutrients needed to calculate NQIs.

Appendix. Details in the Calculation of NQI Values for Specific Foods

The NQI values in the NuCal system reflect the nutrient needs and intakes for women age 19 to 30. The weights calculated for the 27 nutrients are shown in the far-right column in the table below. The steps in the calculation of final index weights are described below the table.

Index Weights for Women Age 19 to 30

A	B	C	D	E	F	G	H	I
27 Nutrients + Calories	RDA	Units	Intake	Intk/RDA	Share	Defic.	Sh*Def	Index Wt.
Calories	2200	calories						
Vitamin A	700	mcg	607	0.87	0.024	1.15	0.028	0.024
Vitamin D	15	mcg	4.6	0.31	0.024	3.26	0.078	0.069
Vitamin E	15	mg	7.2	0.48	0.024	2.08	0.050	0.044
Vitamin K	90	mcg	88.9	0.99	0.024	1.01	0.024	0.021
Vitamin B-6	1.3	mg	1.91	1.47	0.024	0.68	0.016	0.014
Vitamin B-12	2.4	mcg	5.19	2.16	0.024	0.46	0.011	0.010
Vitamin C	75	mg	84.2	1.12	0.024	0.89	0.021	0.019
Folate	400	mcg	527	1.32	0.024	0.76	0.018	0.016
Niacin	14	mg	23.9	1.71	0.024	0.59	0.014	0.012
Riboflavin	1.1	mg	2.16	1.96	0.024	0.51	0.012	0.011
Thiamin	1.1	mg	1.59	1.45	0.024	0.69	0.017	0.015
Calcium	1000	mg	946	0.95	0.024	1.06	0.025	0.022
Potassium	4700	mg	2509	0.53	0.024	1.87	0.045	0.039
Magnesium	310	mg	277	0.89	0.024	1.12	0.027	0.024
Phosphorus	700	mg	1297	1.85	0.024	0.54	0.013	0.011
Copper	0.9	mg	1.3	1.44	0.024	0.69	0.017	0.015
Iron	18	mg	14.7	0.82	0.024	1.22	0.029	0.026
Selenium	55	mcg	104.9	1.91	0.024	0.52	0.013	0.011
Zinc	8	mg	11.6	1.45	0.024	0.69	0.017	0.015
Choline	425	mg	305	0.72	0.024	1.39	0.033	0.029
Fiber	25	grams	15.2	0.61	0.10	1.64	0.164	0.144
Linoleic acid	12	grams	14.5	1.21	0.05	0.83	0.041	0.036
alpha-Linolenic acid	1.1	grams	1.4	1.27	0.05	0.79	0.039	0.034
Protein	46	grams	76.2	1.66	0.16	0.60	0.097	0.085
Lutein+Zeaxanthin	2000	mcg	1362	0.68	0.05	1.47	0.073	0.064
Lycopene	10000	mcg	5219	0.52	0.03	1.92	0.057	0.050
ORAC (total)	20000	µmol TE	10000	0.50	0.08	2.00	0.160	0.140
SUMS					1.000		1.141	1.000

Column A. The 27 nutrients in the index, plus calories
Column B. RDAs, AIs, and estimated AIs used
Column C. Units used for columns B and D
Column D. Average intakes for U.S. women, age 19 to 30, NHANES 2001-2002
Column E. Average intakes as a fraction or multiple of the RDAs (column D/column B)
Column F. Initial share weights for each nutrient (column F sum = 1)
Column G. Average "deficiencies" for U.S. women (1/column E)
Column H. Index weights before normalization (column F × column G)
Column I. Normalized index weights (column H/column H sum) (column I sum = 1)

References

Benbrook, C. 2005. *Elevating Antioxidant Levels in Food through Organic Farming and Food Processing*, Organic Center State of Science Review, access summary and full report at http://www.organic-center.org/science.antiox.php?action=view&report_id=3

Benbrook, C., Zhao, X., Yanez, J., Davies, N., and P. Andrews. 2008. New Evidence Confirms the Nutritional Superiority of Plant-Based Organic Foods, Organic Center State of Science review, access summary in English and Spanish and full report at http://www.organic-center.org/science.nutri.php?action=view&report_id=126

Davis, D.R. 2009. "Declining Fruit and Vegetable Nutrient Composition: What Is the Evidence?," *HortScience* 44:15-19

Davis, D.R., M.D. Epp, and H.D. Riordan. 2004. "Changes in USDA food composition data for 43 garden crops, 1950 to 1999," *J. Amer. Coll. Nutr*. 23:669–682

Drewnowski A. 2005. "Concept of a nutritious food: toward a nutrient density Score," *Am J Clin Nutr*. 82:721–32

Drewnowski, A, and V. Fulgoni, 2007. "Nutrient profiling of foods: creating a nutrient-rich food index," *Nutrition Reviews*, Vol. 66(1):23-29

Fan, M.-S., F.-J. Zhao, S.J. Fairweather-Tait, P.R. Poulton, S.J. Dunham, and S.P. McGrath. 2008. "Evidence of decreasing mineral density in wheat grain over the last 160 years," *J. Trace Elem. Med. Biol*. 22:315-324

Fulgoni III, V.L, D.R. Keast, and A. Drewnowski 2009. "Development and Validation of the Nutrient-Rich Foods Index: A Tool to Measure Nutritional Quality of Foods," *J. Nutr.* 139: 1549–1554

Hansen, R.G. 1973. "An index of food quality," *Nutrition Reviews*, Vol. 31, No. 1.

Hansen, R.G., Wyse, B.W., and A.W. Sorenson. 1979. *Nutrition quality index of food*, Westport Ct: AVI Publishing Co.

Katz, DL, Njike, VY, Kennedy, D. et al., Overall Nutritional Quality Index Version 1 (ONQI.v1)," Prevention research Center, Yale University School of Medicine, Derby, CT. Access at http://www.nuval.com/images/upload/file/ONQI%20Manual%205_5_09.pdf

Miller, G.D., Drewnowski, A., Fulgoni, V., Heaney, R.P., King, J., and E. Kennedy. 2009. "It is Time for a Positive Approach to Dietary Guidance Using Nutrient Density as a Basic Principle," *J. Nutrition*, 139: 1198-1202.

Oxygen Radical Absorbance Capacity (ORAC) of Selected Foods. 2010. Access the USDA's 2010 ORAC report with values for hundreds of foods, and the USDA's ORAC database at: http://www.ars.usda.gov/Services/docs.htm?docid=15866

Strickland Computer Consulting. 2011. *NutriCircles*, access by contacting Roger Strickland, strickla@pacbell.net

U.S. Department of Agriculture. 2009. "Healthy Eating Index," access at http://www.cnpp.usda.gov/healthyeatingindex.htm

U.S. Department of Agriculture, Agricultural Research Service. 2010. *USDA National Nutrient Database for Standard Reference, Release 23*, access at http://www.ars.usda.gov/Services/docs.htm?docid=8964

U.S. Department of Agriculture, and U.S. Department of Health and Human Services, Centers for Disease Control and Prevention, 2007. *What We Eat in America, NHANES 2001-2002. Nutrients Intakes: Mean Amount Consumed per Individual, One Day).* Available from: http://www.swcgrl.ars.usda.gov/Services/docs.htm?docid=18349 [accessed 09/11/06].

Wang SY, Chen CT, Sciarappa W, et al. 2008. "Fruit quality, antioxidant capacity, and flavonoid content of organically and conventionally grown blueberries," *J Agric Food Chem*. 56:5788-5794
